# Parliament and Publications

**Published:** 1910-10-29

**Authors:** 


### Parliament and Publications.

### THE HOSPITALS OF KEDAH.

The annual report of the adviser to the Kedah Government for the year 1327 A.H. (January 23, 1909, to January 12, 1910) contains some information concerning the four hospitals in the State. The total number of admissions to the hospitals during the year was 2,238, as against 2,626 in the preceding year; and there were 133 deaths, as against 261 in the preceding year. The total number of out-patients treated during the year was 24,773. This is the first report written since the transfer of the State of Kedah from the Suzerainty of Siam to that of Great Britain. "Straits Settlements" (Kedah and Perlis). Cd. 5389. Price 9d.

